# Folic acid‐decorated astrocytes‐derived exosomes enhanced the effect of temozolomide against glioma

**DOI:** 10.1002/kjm2.12819

**Published:** 2024-03-14

**Authors:** Hong‐Ming Liu, Ye Zhang

**Affiliations:** ^1^ Shandong Center For Food and Drug Evaluation & Inspection Jinan China; ^2^ Zibo Food and Drug Inspection and Research Institute Zibo China; ^3^ Department of Pharmaceutical Science Zibo Vocational Institute Zibo China

**Keywords:** astrocytes, exosomes, folic acid, glioma, temozolomide

## Abstract

A direct strategy to achieve specific treatments and reduce side effects is through cell type‐specific drug delivery. Exosomes (Exos) can be modified with folic acid (FA) to prepare drug delivery systems targeting tumor cells that highly express FA receptors. This study aimed to produce an exo drug delivery system with FA decoration and temozolomide (TMZ) loading to improve the sustained TMZ release and targeting. We used DSPE‐PEG_2000_‐FA to modify exos derived from astrocyte U‐87 to prepare FA‐modified exos (Astro‐exo‐FA). TMZ was encapsulated into Astro‐exo‐FA or Astro‐exo through electroporation to produce TMZ@Astro‐exo and TMZ@Astro‐exo‐FA. In vitro drug release was examined using the dialysis bag method. Through cell experiments in vitro and mouse glioma models in vivo, the effect of TMZ@Astro‐exo‐FA on U‐87 cells was determined. Exo properties were not affected by FA modification and TMZ loading. The drug release rate of TMZ@Astro‐exo‐FA was slower. TMZ@Astro‐exo‐FA uptake by U‐87 cells was higher compared to TMZ@Astro‐exo, indicating that TMZ@Astro‐exo‐FA has a stronger targeting toward U‐87 cells. TMZ@Astro‐exo‐FA remarkably reduced U‐87 cell proliferation, migration, and invasion compared with TMZ@Astro‐exo and free TMZ. Treatment with TMZ@Astro‐exo‐FA reduced the side effects of TMZ (minimal change in body weight), prolonged survival, and inhibited tumor growth in mouse glioma models, and its efficacy was stronger than that of TMZ@Astro‐exo and free TMZ. TMZ@Astro‐exo‐FA could enhance the effect of TMZ against glioma, providing novel ideas for drug targeting delivery and exploring exos as drug carriers against glioma.

## 1 INTRODUCTION

Glioma, a malignant intracranial tumor that occurs in the neuroectoderm, accounts for approximately 40%–50% of craniocerebral tumors.^1^ Presently, surgical resection, radiotherapy, and chemotherapy or in combination are the main treatment strategies for glioma.^2^ The characteristic infiltrative growth of glioma blurs the boundary between normal brain tissue and tumor tissue, leading to difficulty in complete tissue removal through surgical treatment, rendering a poor treatment effect with a high risk of recurrence.^3^, ^4^

Temozolomide (TMZ) is a first‐line drug for the clinical treatment of brain tumors, with excellent properties such as lipophilicity, acid stability, less toxic side effects, and ability to penetrate the blood–brain barrier (BBB).^5^, ^6^ TMZ is hydrolyzed at physiological pH ≥7.4, releasing a methyldiazonium cation to methylate purine bases (O6‐guanine, N7‐guanine, and N3‐adenine), stimulating anti‐tumor activity.^7^ However, TMZ does not possess specific targeting to glioma cells and is unstable, making it prone to degradation under human environmental conditions; thus, clinically, frequent administration is required to maintain an effective TMZ concentration.^7^, ^8^ Importantly, frequent administration not only leads to low drug utilization, but also causes serious damage to normal cells.^9^, ^10^ Hence, the development of TMZ carriers with specific targeting has become a new research hotspot.

Exosomes (exos) are membranous small vesicles released into the extracellular matrix by different types of cells, which are widely present in cell culture supernatants and various body fluids, with a diameter of approximately 30–150 nm.^11^ Biologically active substances can be transported from donor cells to recipient cells through exos, allowing communication between cells.^12^ Exo‐mediated delivery can circumvent the efflux system of P‐glycoprotein drugs, thereby reducing drug resistance.^13^ Furthermore, the circulating stability of exos can impede the phagocytosis of macrophages and prolong the half‐life of chemical drugs.^14^, ^15^ Concurrently, exos can also undergo genetic engineering processing, and by modifying the surface proteins of exos, they can specifically bind to receptors on the target cell surface, thereby making exo‐mediated transportation with cell and tissue specificity.^16^ Additionally, in clinical applications, the high biocompatibility and low toxicity of naturally derived exos render them superior to synthetic drug carriers.^17^ Thus, in the treatment of glioma, exos as effective anti‐cancer drug carriers have attracted widespread attention.

Folic acid (FA) is a small‐molecular‐weight vitamin. FA receptors are membrane glycoproteins that are highly expressed on some tumor cell surface.^18^ Therefore, FA can be used to modify the exos carrying the drug, causing engineered exos to have specific targeting properties, exerting a killing effect on tumor cells and reducing normal tissue damage.^19^, ^20^ FA‐functionalized exos co‐loaded with resveratrol and celastrol demonstrated powerful anti‐inflammatory and immunosuppressive activities against LPS‐stimulated macrophages in vitro.^21^ An exo‐based drug delivery system expressing PH20 and modifying FA could enhance the tumor treatment efficiency and reduce the side effects of hyaluronidase treatment.^22^ Therefore, taking advantage of FA receptor overexpression in glioma cells, the FA‐decorated exos loaded with TMZ can actively target TMZ‐loaded exos to glioma cells with FA receptors as targets, thereby improving the distribution of TMZ‐loaded exos in tumor tissues and achieving the aim of the targeted therapy. We mainly constructed FA‐modified TMZ‐loaded astrocyte‐derived exos (TMZ@Astro‐exo‐FA) and evaluated the effect of TMZ@Astro‐exo‐FA on astrocytes through a series of cell experiments in vitro and glioma mouse models in vivo, providing a direction for the development of new drug carriers that can target glioma.

## 2 MATERIALS AND METHODS

### 2.1 Glioma cells

The U‐87 cells (Cat#HTB‐14, ATCC, Manassas, VA, USA) were cultured in Dulbecco's modified Eagle's medium (DMEM) (Cat#D5796, Sigma, St. Louis, MO, USA) supplemented with 10% fetal bovine serum (FBS) (Cat#26010074, Gibco, Thermo, Waltham, MA, USA) and 1% penicillin/streptomycin (Cat#15140122, Gibco) in a standard humidified incubator at 37°C under 5% CO_2_. For the extraction of exos from cell culture supernatant, exo‐depleted FBS media supplement (Cat#EXO‐FBSHI‐250A‐1SBI, Palo Alto, CA, USA) was utilized.

### 2.2 Isolation of U‐87 cell‐derived exos

Ultracentrifugation is the most frequently employed method for the isolation of exos.^23^ For exo isolation, the cell culture supernatants containing exos were harvested after a 48‐h culture. The obtained supernatant was first centrifuged (300*g*, 10 min) (L‐80XP ultracentrifuge, Beckman Coulter, Brea, CA, USA) to eliminate dead cells, followed by re‐centrifugation (2000 g, 10 min) to remove cellular debris. Centrifugation was again performed at 10,000 g for 30 min to remove large vesicles. Subsequently, centrifugation (120,000 g) was conducted for 90 min to aggregate the pellet. The obtained pellets were then resuspended in cold PBS, followed by filtration via a 0.22‐μm filter. Finally, centrifugation (120,000 g) was performed for 90 min to obtain exos. The quantification and analysis of the obtained exo fraction were performed through quantitative protein estimation using the BCA protein assay kit (Cat#P0011, Beyotime, Shanghai, China). Typical exo markers CD63, CD81, and Tsg101 were analyzed using western blotting. The diluted exos were then applied to a copper grid for 1 min, followed by staining with 1% uranyl nitrate for 30 s. The Tecnai G2 Spirit transmission electron microscope (TEM) was used to examine the dried grids, operating at 120 kV. The size distribution and particle concentration were analyzed and recorded using the NanoSight NS300 system (Malvern, UK) and Nanoparticle Tracking Analysis software (NTA, version 2.3).

### 2.3 Production of engineered TMZ@Astro‐exo and TMZ@Astro‐exo‐FA

The isolated exos (50 μg/mL, total protein) were co‐incubated with the DSPE‐PEG_2000_‐FA (50 μg/mL) for 30 min with shaking at 37°C to prepare Astro‐exo‐FA. TMZ was loaded into the Astro‐exo‐FA through electroporation to obtain TMZ@Astro‐exo‐FA, according to a previous study.^24^ Thus, TMZ (200 μg) and exos (200 μg) were mixed with PBS to a final volume of 500 μL in an electroporation cuvette. The mixture was electroporated at 500 V with one discharge for 1 ms. The suspension was incubated at 4°C for 15 min after electroporation, followed by a continued incubation for 1 h at 37°C to promote drug diffusion and pore closure. Finally, the mixture was centrifuged twice (4000*g*, 30 min) in 100‐KDa ultrafiltration tubes to remove extra‐exosomal TMZ. Consequently, the characterization of TMZ@Astro‐exo and TMZ@Astro‐exo‐FA was analyzed using NTA, TEM, and western blotting.

### 2.4 Western blotting

The exosomal markers CD63, CD81, and Tsg101 were confirmed through western blotting. In brief, radioimmunoprecipitation assay buffer (Cat#R0020, Solarbio, Beijing, China) supplemented with PMSF was used to lyse U‐87 cells, TMZ@Astro‐exo, and TMZ@Astro‐exo‐FA. Protein samples were electrophoresed with 10% sodium dodecyl‐sulfate polyacrylamide gel electrophoresis and then were transferred onto polyvinylidene difluoride membranes (Sigma). After blocking with 5% non‐fat dry milk, the membrane was incubated with primary antibody against CD63, CD81, Tsg101 or β‐actin at 4°C overnight. All information on all antibodies is presented in Table S1. The PVDF membranes were visualized with a gel imaging system after incubating with HRP‐conjugated secondary antibody (Millipore, Bedford, MA, USA).

### 2.5 Analysis of drug release

The release rate of free TMZ, TMZ@Astro‐exo, and TMZ@Astro‐exo‐FA was evaluated using PBS (pH 7.4) as the release medium. The release medium (25 mL) was used for the immersion of a sealed dialysis bag with TMZ@Astro‐exo and TMZ@Astro‐exo‐FA (5 μL). Equal amounts of fresh release medium were added after collecting the release medium (500 μL). Each sample was measured at 254 nm through high‐performance liquid chromatography (Shimadzu LC‐2030, Japan).

### 2.6 PKH26‐labeled exos and their endocytosis

For the intracellular uptake assay, the isolated exos were labeled with PKH‐26 (red) (Cat#D0030, Solarbio) as per manufacturer's protocol. The U‐87 cells (1 × 10^4^ cells) were incubated for 1 and 2 h with PKH‐labeled exos (30 μg) dispersed in basal DMEM. After removing DMEM, cells were washed thrice to remove un‐internalized exos. Post‐washing cells were fixed with paraformaldehyde (PAF) (4% w/v) (Cat#P1110, Solarbio) for 30 min and then incubated in Triton‐X 100 (0.5% v/v) (Cat#IT9100, Solarbio) for 20 min. The cells were washed with PBS and stained with DAPI (Cat#C1005, Beyotime) after incubation. Observation of the cells was conducted using a confocal fluorescence microscope (IX73, Olympus, Tokyo, Japan).

### 2.7 Cell counting kit‐8 (CCK‐8) assay

The U‐87 cells (1 × 10^4^ cells) were seeded in 96‐well plates for cell viability analysis. After 24 h, 0.05% dimethyl sulfoxide (DMSO) (control) (Cat#ST038, Beyotime), TMZ, TMZ@Astro‐exo, or TMZ@Astro‐exo‐FA were respectively added and co‐cultured for 1–3 days. Subsequently, 10‐μL CCK‐8 (Cat#KGA317, KeyGen Biotech, Nanjing, China) was added to each pore. After a 3‐h incubation, the absorbance value (OD 450 nm) was colorimetrically determined (Multiskan MK3, Thermo). All cell lines were plated at 2.0 × 10^3^ cells/well and treated with a series of doses of free TMZ or TMZ@Astro‐exo‐FA 24 h later. At 24 h after treatment, IC50 values were calculated according to cell viability as determined by the CCK‐8 assay.

### 2.8 5‐ethynyl‐2′‐deoxyuridine (EdU) fluorescent staining

To assess cell proliferation, EdU fluorescent staining was performed. A total of 1.0 × 10^4^ U‐87 cells were seeded in 24‐well plates containing 0.05% DMSO (control), TMZ, TMZ@Astro‐exo, or TMZ@Astro‐exo‐FA. After a 24‐h incubation, the medium was discarded, and the medium containing 50 μmol/L EdU (Cat#C10310‐3, RiboBio, Guangzhou, China) was added to each well. The cells were fixed with PAF (4% w/v) 24 h later, followed by staining with Apollo and Hoechst. The images were photographed under a fluorescence microscope. The number of EdU‐positive cells was calculated as the percentage of total cells per random field.

### 2.9 Clonogenic assay

The clonogenic assay is an in vitro survival assay based on the ability of a single cell to grow into a colony. After harvesting with 0.05% trypsin, 1 × 10^3^ cells were plated in six‐well plates with 0.05% DMSO (control), TMZ, TMZ@Astro‐exo, or TMZ@Astro‐exo‐FA. To allow colony formation, cells were incubated for 15 days after the treatment. Colonies were fixed with methanol and stained with 1% crystal violet.

### 2.10 Wound‐healing assay

Briefly, the U‐87 cells were seeded in a complete medium of 6‐well plates until a more than 80% confluency is attained. A wound was made in each monolayer using a 20‐μL pipette tip. The cells were incubated for 48 h after removing the floating cells. Images were captured using an inverted microscope at 0 and 48 h and quantified using TScratch software.

### 2.11 Transwell invasion assay

Briefly, the U‐87 cells were added into 24‐well plates with an 8‐μm‐pore polycarbonate membrane (Cat#3422, Corning, NY, USA) coated with 20 mg of Matrigel (Cat#354230, BD Biosciences). Approximately 2 × 10^4^ cells were cultured in the upper chamber in serum‐free medium. Furthermore, a medium containing 10% FBS was added to the lower chamber. After a 24‐h incubation, the invasive cells were fixed using 4% PFA, stained using 0.1% crystal violet (Cat#C0121 Beyotime) for 30 min, and photographed using an inverted microscope. Three independent experiments were conducted.

### 2.12 In vivo anti‐tumor efficacy

Mice were housed at 22°C in a 12‐h light/12‐h dark cycle in a specific pathogen‐free animal house. All animal experiments were conducted in accordance with the guidelines provided by Zibo Vocational Institute. The orthotopic U‐87 mouse model was established in female NCG (NOD/ShiLtJGpt‐Prkdc^em26^Il2rg^em26^/Gpt) mice (Jicuiyaokang, Jiangsu, China). U‐87 cells (1 × 10^6^ cells) were suspended in PBS (1 μL) and implanted into mice brainstem as previously described operation steps.^25^ After a 6‐day cellular implantation, mice were randomly assigned into four groups (*n* = 6) and treated with PBS, TMZ, TMZ@Astro‐exo, or TMZ@Astro‐exo‐FA (5 mg/kg) to evaluate the therapeutic efficacy via the tail vein. The treatment was repeated every 3 days for a total of four doses. The body weight of mice was recorded every 3 days during therapy. On day 21, one mouse in each group was randomly euthanized to remove glioma for hematoxylin–eosin staining (H&E). The remaining mice were retained until they died for survival analysis. Blood was collected from each mouse for serum chemistry analysis.

### 2.13 Statistical analysis

GraphPad Prism v8.0 (GraphPad Inc., La Jolla, CA, USA) was used for all statistical analyses. Data were evaluated for normality using the Kolmogorov–Smirnov test. Experimental data are presented as the mean ± standard deviation. A comparison of the two groups was executed using a two‐sided Student's *t*‐test. Multiple comparisons were conducted using one‐way analysis of variance plus a two‐sided Tukey's test. Survival curves were plotted using the Kaplan–Meier method and compared using log‐rank tests. All tests were two‐tailed and statistical significance was set at *p* < 0.05.

## 3 RESULTS

### 3.1 Preparation of TMZ@Astro‐exo‐FA

**FIGURE 1:**
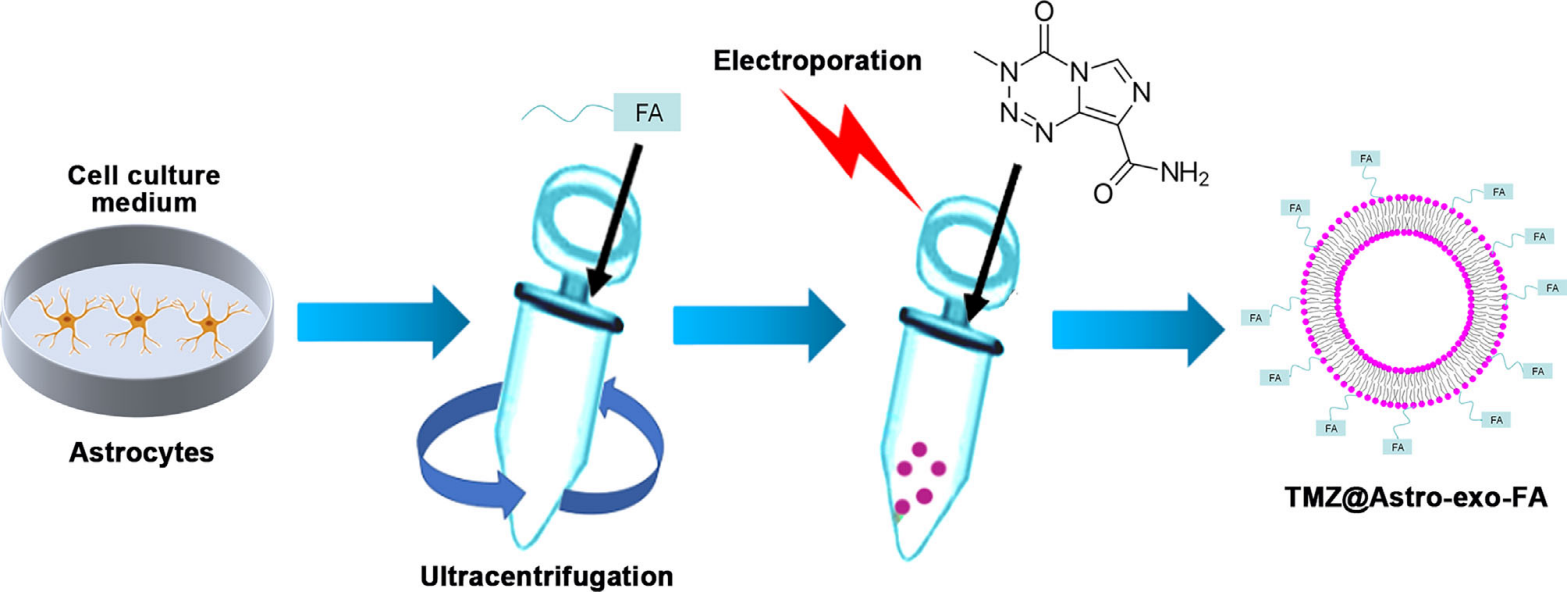
Schematic diagram representing the generation of engineered exos TMZ@Astro‐exo‐FA.

The TMZ@Astro‐exo‐FA was prepared by following the procedures as illustrated in Figure 1. Exos were isolated from astrocyte U‐87 through ultracentrifugation and then coated with the membrane‐insertable FA derivative of DSPE‐PEG_2000_‐FA through its hydrophobic lipid tail. Loading of TMZ into Astro‐exo‐FA was achieved through an electroporation approach, and the resultant formulation was termed TMZ@Astro‐exo‐FA for brevity.

### 3.2 TMZ@Astro‐exo‐FA morphology and characterization

**FIGURE 2:**
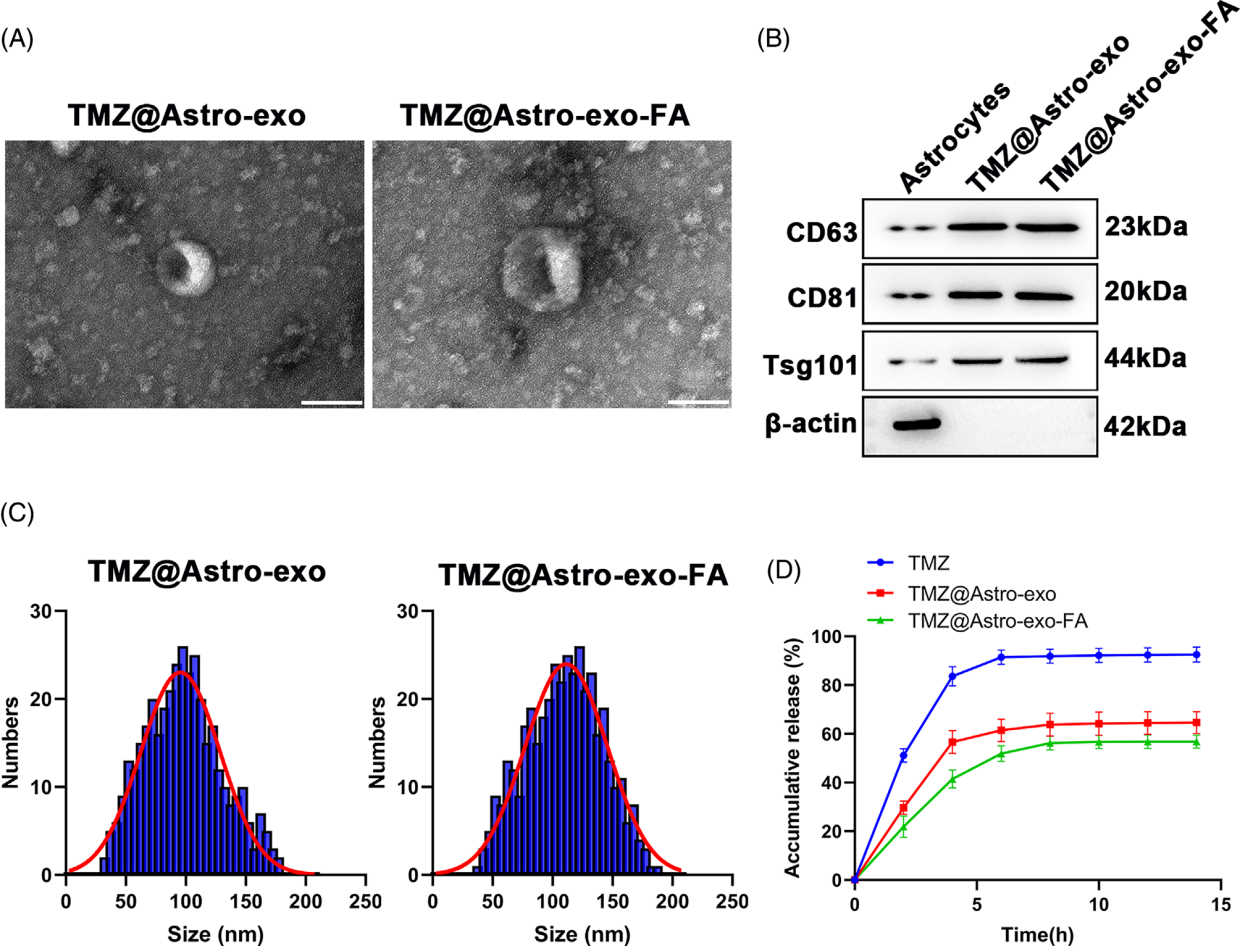
TMZ@Astro‐exo‐FA characterization. (A) Representative TME images for TMZ@Astro‐exo and TMZ@Astro‐exo‐FA. (B) Characteristic membrane proteins of TMZ@Astro‐exo and TMZ@Astro‐exo‐FA were analyzed through western blotting. (C) Particle size distributions of TMZ@Astro‐exo and TMZ@Astro‐exo‐FA were measured using NTA. (D) In vitro release curves of TMZ@Astro‐exo and TMZ@Astro‐exo‐FA in PBS.

TMZ@Astro‐exo and TMZ@Astro‐exo‐FA morphology was further characterized using TEM. We observed that both TMZ@Astro‐exo and TMZ@Astro‐exo‐FA were round and cup‐shaped vesicles with a bilayer membrane structure (Figure 2A). Western blotting demonstrated that both TMZ@Astro‐exo and TMZ@Astro‐exo‐FA expressed exo marker proteins CD63, CD81, and Tsg101 (Figure 2B). NTA results revealed that the main peak of the TMZ@Astro‐exo diameter was 100 nm, while the main peak of the TMZ@Astro‐exo‐FA diameter was 110 nm. Although the particle size of TMZ@Astro‐exo‐FA had a minimal increase, it still conformed to the characteristics of exos, suggesting that the properties of exos were not affected by FA decoration and drug loading (Figure 2C). The in vitro release of TMZ, TMZ@Astro‐exo, and TMZ@Astro‐exo‐FA was explored through a dialysis bag method. The free TMZ showed a faster release rate, with a cumulative release rate of up to 80% at 4 h. Differently, TMZ in TMZ@Astro‐exo and TMZ@Astro‐exo‐FA was wrapped by exos, making TMZ release slower and smoother, with a cumulative release increasing to approximately 60% and 50% from 0 to 10 h, respectively (Figure 2D). These results manifested that TMZ@Astro‐exo‐FA did not alter exo properties and prolonged drug release in vitro.

### 3.3 TMZ@Astro‐exo‐FA enhanced TMZ toxicity to astrocytes

**FIGURE 3:**
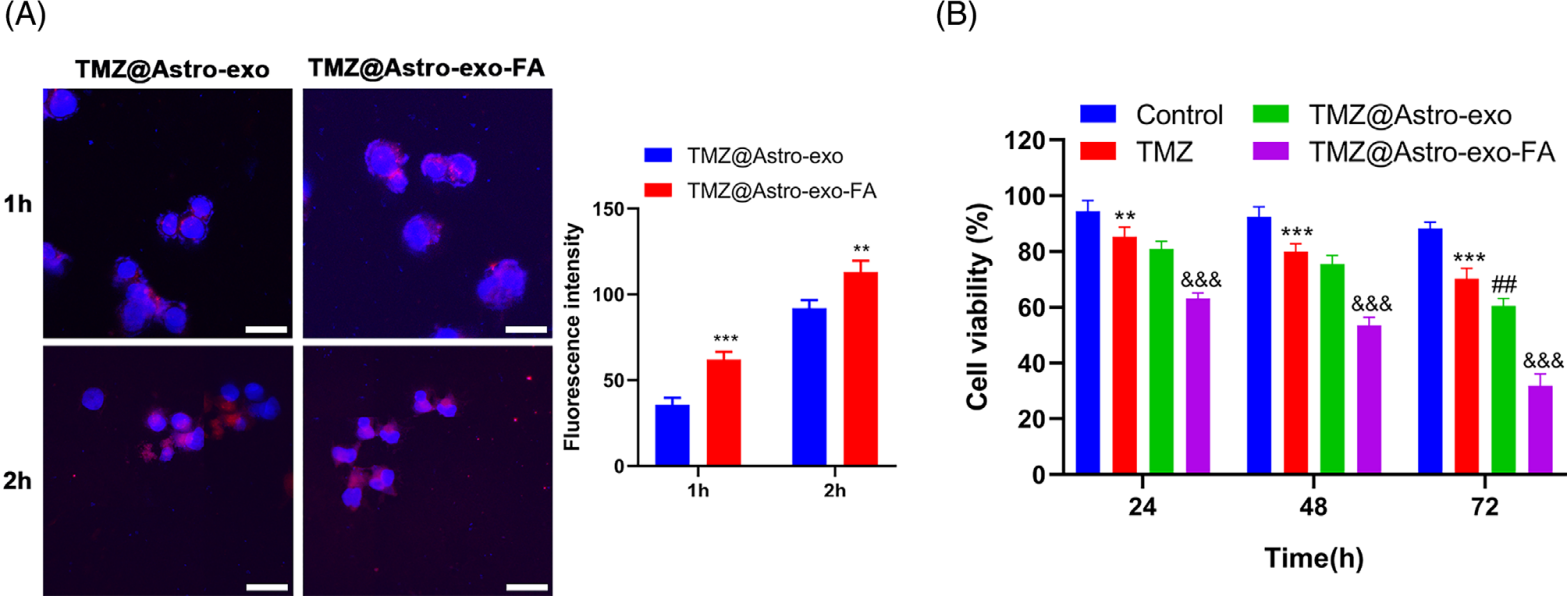
Targeting ability and cytotoxicity of TMZ@Astro‐exo‐FA. (A) Confocal microscopy images of U‐87 cells incubated with PKH26‐TMZ@Astro‐exo or TMZ@Astro‐exo‐FA and its quantitative data. **p < 0.01 and ***p < 0.001 versus TMZ@Astro‐exo. (B) The toxicity of control (0.05% dimethyl sulfoxide [DMSO]), TMZ, TMZ@Astro‐exo, and TMZ@Astro‐exo‐FA on U‐87 cells was determined using CCK‐8 assays. **p < 0.01 and ***p < 0.001 versus control; ^##^ *p* < 0.01 versus TMZ; ^&&&^ *p* < 0.001 versus TMZ@Astro‐exo. Data are expressed as means ± standard deviation (*n* = 3).

TMZ@Astro‐exo and TMZ@Astro‐exo‐FA were first labeled with the membrane dye PKH26 (red) and then co‐cultured with U‐87 cells to demonstrate that FA modification can improve the binding capability of Astro‐exo to astrocytes. After incubation for 1 and 2 h, two types of exos (TMZ@Astro‐exo and TMZ@Astro‐exo‐FA) were successfully detected in the recipient U‐87 cells, and U‐87 cells in the TMZ@Astro‐exo‐FA group had a considerably higher PKH26 fluorescence signal than the TMZ@Astro‐exo group (Figure 3A). Next, the toxicity of TMZ@Astro‐exo‐FA to U‐87 cells was analyzed. Astrocytes were treated with solvent DMSO, free TMZ, TMZ@Astro‐exo, and TMZ@Astro‐exo‐FA for different hours. We noted that TMZ demonstrated light cytotoxicity on astrocytes versus DMSO at 24, 48, and 72 h, while TMZ@Astro‐exo had a stronger cytotoxicity versus free TMZ at 72 h. Notably, TMZ@Astro‐exo‐FA showed a significantly stronger cytotoxicity than TMZ@Astro at 24, 48, and 72 h (Figure 3B), confirming that the TMZ@Astro‐exo‐FA had a significant cytotoxicity to astrocytes in vitro. In line with the abovementioned results, TMZ@Astro‐exo‐FA resulted in a two‐ to threefold increase in cell death as indicated by changes in the IC50 values compared to free TMZ in three types of glioma cell lines (Supplementary Table S2). To investigate the specificity and sensitivity of TMZ@Astro‐exo‐FA to glioma, the potential of TMZ@Astro‐exo‐FA to kill various types of non‐glioma cancers in vitro was evaluated. However, no significant difference was found between the efficiency of TMZ and TMZ@Astro‐exo‐FA in non‐glioma cancers (Supplementary Table S2). These results indicate that the potential of Astro‐exo‐FA to enhance anti‐tumor effect of TMZ might be limited to glioma.

### 3.4 TMZ@Astro‐exo‐FA strengthened TMZ‐mediated inhibitory effects on astrocyte proliferation, migration, and invasion

**FIGURE 4:**
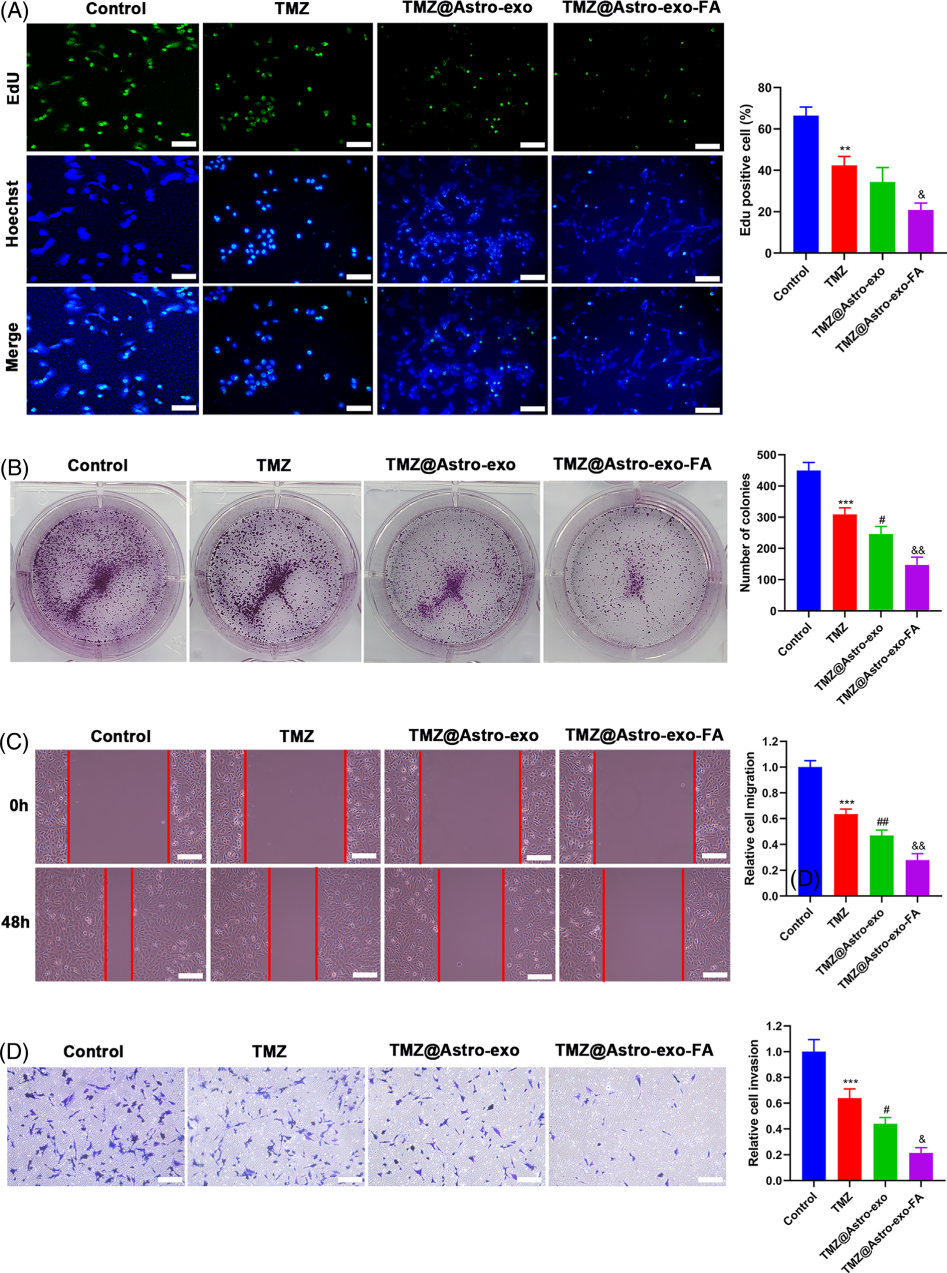
TMZ@Astro‐exo‐FA increased the inhibiting effect of TMZ on U87 cell proliferation, migration, and invasion. (A–D) Effects of control (0.05% DMSO), TMZ, TMZ@Astro‐exo, and TMZ@Astro‐exo‐FA on U‐87 cell proliferation, migration, and invasion were evaluated using EdU (A), clonogenic (B), wound‐healing (C), and Transwell invasion assays (D). **p < 0.01 and ***p < 0.001 versus control; ^#^ *p* <  0.05 and ^##^ *p* < 0.01 versus TMZ; ^&^ *p* < 0.05 and ^&&^ *p* < 0.01 versus TMZ@Astro‐exo. Data are expressed as means ± SD (*n* = 3).

he effects of TMZ@Astro‐exo‐FA on astrocyte proliferation, migration, and invasion were further evaluated. After 24 h of incubation, TMZ resulted in a significant decrease in the percentage of EdU‐positive U‐87 cells (to 40%) versus DMSO, while TMZ@Astro‐exo‐FA caused an overt reduction in the percentage of EdU‐positive U‐87 cells (to 20%) compared to TMZ@Astro‐exo, as validated by the EdU assays (Figure 4A). The clonogenic assay conducted in U‐87 cells showed that TMZ repressed the colony formation ability of U‐87 cells. However, TMZ@Astro‐exo exhibited a stronger inhibitory effect on the colony formation ability of U‐87 cells than free TMZ, but weaker than TMZ@Astro‐exo‐FA. Similarly, after TMZ treatment, the migrating and invading capacities of U‐87 cells were impaired. However, the inhibitory effect of TMZ@Astro‐exo on the U‐87 cell behaviors was greater than that of free TMZ, but less than TMZ@Astro‐exo‐FA. Altogether, these findings indicated that TMZ@Astro‐exo‐FA strengthened TMZ‐mediated inhibitory effects on astrocyte proliferation, migration, and invasion.

### 3.5 TMZ@Astro‐exo‐FA improved the efficacy of TMZ on glioma in mouse models

**FIGURE 5:**
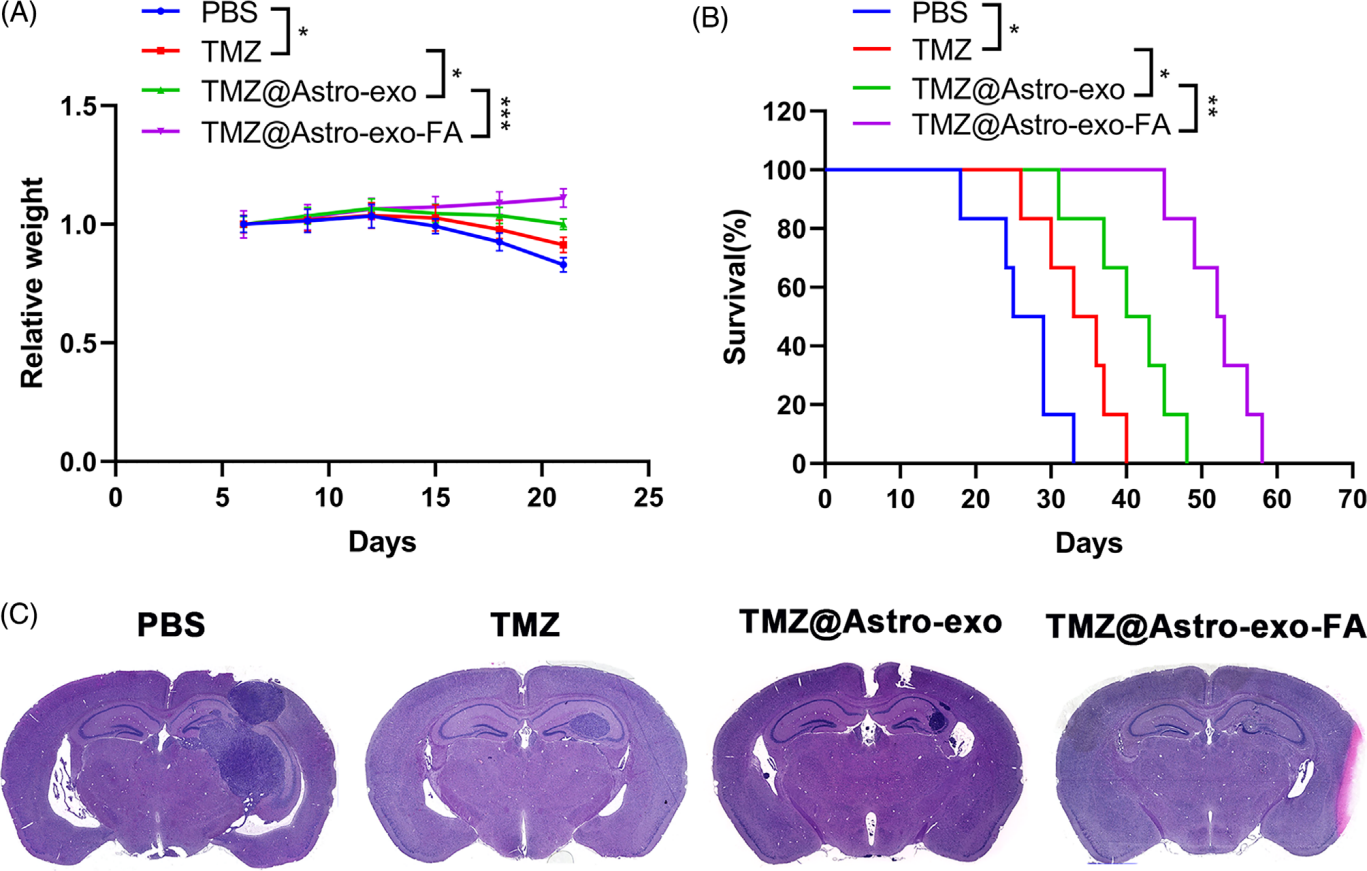
TMZ@Astro‐exo‐FA enhanced the effect of TMZ against glioma in mouse orthotopic xenograft models. (A) Body weight changes of mouse glioma models in PBS, TMZ, TMZ@Astro‐exo, and TMZ@Astro‐exo‐FA groups (*n* = 6). (B) Kaplan–Meier survival curves of mouse glioma models in different treatment groups. (C) Hematoxylin–eosin staining images of brain tissues derived from different treatment groups. *p < 0.05, **p < 0.01, and ***p < 0.001.

We injected U‐87 cells into the brains of nude mice to establish an intracranial orthotopic xenograft model to clarify the targeting ability of TMZ@Astro‐exo‐FA in glioma in vivo. Mice were randomly assigned to four groups, followed by PBS, TMZ, TMZ@Astro‐exo, and TMZ@Astro‐exo‐FA administration (TMZ administered at a concentration of 5 mg/kg) through the tail vein, with injections every 3 days for four consecutive doses. Body weight changes in mice treated with different drugs were recorded. Only mice in the TMZ@Astro‐exo‐FA group did not have a significant decline in body weight, while mice in the remaining groups had a significant decrease in body weight with the following ranking: TMZ@Astro‐exo‐FA > TMZ@Astro‐exo > TMZ > PBS (Figure 5A). Additionally, mice in the PBS group had the shortest survival time, which is attributed to the rapid growth of gliomas in mice in this group. In other groups, tumor growth was inhibited to varying degrees secondary to drug treatment, resulting in the prolonged survival time of mice, and the survival time ranking was TMZ@Astro‐exo‐FA > TMZ@Astro‐exo > TMZ > PBS. Most importantly, mice in the TMZ@Astro‐exo‐FA group had the longest survival time, which highlighted the excellent anti‐tumor therapeutic effect of TMZ@Astro‐exo‐FA (Figure 5B). H&E staining revealed that brain tissues derived from mice in the TMZ@Astro‐exo‐FA group had the smallest tumor volume, and the ranking of brain tumor volumes in mice in four groups was as follows: TMZ@Astro‐exo‐FA < TMZ@Astro‐exo < TMZ < PBS (Figure 5C). These results indicate that TMZ@Astro‐exo‐FA could effectively improve the effect of TMZ in mouse models. Finally, to investigate whether Astro‐exo‐FA can reduce the toxicity of TMZ on the liver and kidneys of mouse model, we collected blood samples after finishing treatment and examined related parameters. Liver enzyme elevation (alanine transaminase and aspartate aminotransferase) and blood urea nitrogen were evident after treatment with free TMZ, but not with TMZ@Astro‐exo‐FA and TMZ@Astro‐exo (Supplementary Table S3). These results show that TMZ encapsulation in the Astro‐exo‐FA has the potential to reduce the incidence of liver and kidney injury induced by TMZ.

## 4 DISCUSSION

As a first‐line drug for the treatment of glioma, TMZ can effectively cross the BBB to exert cytotoxic effects^26^; however, its application is limited owing to its low lipophilicity and water solubility, including a short half‐life in vivo. In recent years, the application of nanotechnology in the preparation of sustained‐release targeted formulations of various drugs has developed rapidly.^27^ Researchers have utilized nanodrug delivery systems to heighten TMZ solubility, achieve targeted delivery, prolong circulation time in vivo, and reduce adverse drug reactions by encapsulating TMZ in liposomes, polymer micelles, or nanoparticles.^28^ For example, Zong et al. prepared angiopep 2‐modified lipid‐polymer nanoparticles for TMZ delivery to achieve synergistic effects against glioma.^29^ A delivery system of polyhedral oligosesesquitoxanes modified by FA and iRGD peptides shuttled TMZ to glioma cells, effectively enhancing the therapeutic effect of chemotherapy.^30^ Eudragit® RS100 and chitosan‐coated TMZ‐loaded selenium nanoparticles improved the ability to target the delivery of TMZ to C6 cells and reduced protein levels associated with TMZ resistance.^31^ However, synthetic nanomaterials are difficult to degrade and easy to remove using the reticuloendothelial system, which are a disadvantage. Moreover, the TMZ nanodrug delivery systems need to overcome barriers such as blood, tumor cells, and intracellular transport after administration, which limits the efficacy of targeted delivery.

Exo is a natural carrier produced by endogenous cells, with excellent properties such as good biocompatibility, avoidance of phagocytosis by macrophages, blood vessel penetration, and capacity for crossing the BBB.^32^ The disadvantage of insufficient targeting of natural exos can be overcome through reasonable engineering modifications.^16^ A report revealed that glioma cell‐derived exos carrying TMZ and dihydrotanshinone have a variety of advantages, including optimal BBB penetration, tumor‐homing accumulation, and immune response stimulation.^33^ Liang et al. prepared a delivery system by which angiopep‐2 and CD133 RNA aptamers‐decorated exos loaded with O^6^‐benzylguanine and TMZ exhibited efficient uptake by U‐87 cells and excellent extension of the survival time of U‐87‐bearing mice.^34^ However, the efficacy of such delivery system not only hinges on their ability to target tumors efficiently, they must be able to avoid effects of systemic drug toxicity and adverse off‐target tissue effects. Surface modification of nanodelivery system with PEG can increase circulation time, allowing for tumor site accumulation, and minimizing adverse drug toxicities, which is observed with the PEGylated liposomal formulation of doxorubicin that significantly reduces doxorubicin cardiotoxicity.^35^ DSPE‐PEG_2000_‐FA is an amphiphilic linear polymer, with one end of the DSPE chain possessing lipophilicity and the other end possessing hydrophilicity.^36^ In the process of DSPE‐PEG_2000_‐FA modifying exos, the DSPE end can bind to phospholipid membranes and expose FA as a target tip to the surface of exos, which not only increases the in vivo circulation time of exos, but also targets lesions with high FA receptor expression.^37^ Here, DSPE‐PEG_2000_‐FA to modify exos derived from astrocyte U‐87 for the preparation of Astro‐exo‐FA were used. TMZ was encapsulated into Astro‐exo‐FA or Astro‐exo by electroporation to prepare TMZ@Astro‐exo and TMZ@Astro‐exo‐FA. TMZ@Astro‐exo and TMZ@Astro‐exo‐FA maintained the morphological structure of the original exos; however, the particle size was enlarged from 100 to 110 nm after FA modification, indicating that exo properties were not affected by FA modification and TMZ loading. The drug release rate in TMZ@Astro‐exo and TMZ@Astro‐exo‐FA was slower compared with free TMZ, suggesting that exo inclusion has some sustained‐release effect, and the TMZ@Astro‐exo‐FA released the drug at a slower rate than TMZ@Astro‐exo. TMZ@Astro‐exo‐FA was taken up more by target cells after co‐incubation of TMZ@Astro‐exo and TMZ@Astro‐exo‐FA with U‐87 cells, which may be related to the strong affinity of U‐87 cells for TMZ@Astro‐FA due to the high expression of FA receptors. These results exhibit that the TMZ@Astro‐exo‐FA drug delivery system has properties of sustained drug release and astrocyte targeting.

Further experiments on U‐87 cells in vitro showed that TMZ@Astro‐exo‐FA had a stronger inhibitory effect on proliferation, migration, and invasion of U‐87 cells than TMZ@Astro‐exo, while TMZ@Astro‐exo had a stronger inhibitory effect than free TMZ. Kim et al. have developed a tumor‐targeting immunoliposome nanocomplex, which significantly enhanced the efficacy of TMZ in killing a variety of tumor cells (including glioblastoma multiforme and non‐glioblastoma multiforme).^38^ In our study, Astro‐exo‐FA has no significant effect in enhancing the toxicity of TMZ on various types of non‐glioma cancers. This suggests the specificity of Astro‐exo‐FA to glioma. In vivo mouse glioma models showed that mice treated with TMZ@Astro‐exo‐FA had smaller brain tumors and longer survival times than mice treated with TMZ@Astro‐exo, and the treatment efficacy of free TMZ was worse than that of TMZ@Astro‐exo‐FA and TMZ@Astro‐exo. TMZ@Astro‐exo‐FA delivery systems have a natural targeting effect, which can reduce being captured by the reticuloendothelial system,^39^ prolong the drug's half‐life, and deliver TMZ targeting to tumor tissue to ascertain tumor site accumulation, showing a better inhibitory effect on the tumor.

This study has limitations. In the future, animal experiments with expanded samples and delayed observation time are warranted to assess the dose effects, biodistribution, and pharmacokinetics of TMZ@Astro‐ exo‐FA. Second, only one glioma cell line was used in this study, and more glioma cells (such as C6 and GL261) are needed to further confirm the capacity of TMZ@Astro‐exo‐FA on glioma in vivo. Third, the production yield of TMZ@Astro‐exo‐FA is low (2 μg protein/day/10^6^ cells) compared to the need for exos (10 μg per mouse in the present study). Hence, another obstacle that should be overcome in the future is how to get higher production yields of TMZ@Astro‐exo‐FA for practical cell‐free therapy.

In conclusion, TMZ@Astro‐exo‐FA could enhance the effect of TMZ against glioma. The exos drug delivery system prepared in this study will provide novel ideas for drug targeting delivery and exploring exos as drug carriers against glioma.

## CONFLICT OF INTEREST STATEMENT

The authors declare no conflict of interest.

## Supporting information


**Table S1.** The information of antibodies used in western blotting.

**Table S2.** Sensitivity of glioma and other non‐glioma tumor cells by TMZ and TMZ@Astro‐exo‐FA.

**Table S3.** The biochemical parameters in mice after different treatments values are mean ± SD for six mice in each group. ALP, alkaline phosphatase; ALT, alanine transaminase; AST, aspartate aminotransferase; BUN, blood urea nitrogen; Cre, creatinine. (L) represents a lower than 25% change from the untreated baseline, and (H) represents higher than 25% change from the untreated baseline.
