# Supplementary material for: Folic acid‐decorated astrocytes‐derived exosomes enhanced the effect of temozolomide against glioma
Source: Kaohsiung J Med Sci. 2024 Mar 14;40(5):435–44. doi: 10.1002/kjm2.12819 (PMC11895587; doi:10.1002/kjm2.12819)
Supplement: Supplementary file 1 — Table S1. The information of antibodies used in western blotting. [file KJM2-40-435-s003.docx]

**Table S1 The information of antibodies used in western blotting**

| Antibody | Manufacturers | Cat.no | molecular weight |
| --- | --- | --- | --- |
| CD63 | Beyotime | AF1471 | 23kDa |
| CD81 | Beyotime | AG1530 | 20kDa |
| Tsg101 | Beyotime | AF8259 | 44kDa |
| β-actin | Beyotime | AF5003 | 42kDa |
