# Supplementary material for: Folic acid‐decorated astrocytes‐derived exosomes enhanced the effect of temozolomide against glioma
Source: Kaohsiung J Med Sci. 2024 Mar 14;40(5):435–44. doi: 10.1002/kjm2.12819 (PMC11895587; doi:10.1002/kjm2.12819)
Supplement: Supplementary file 2 — Table S2. Sensitivity of glioma and other non‐glioma tumor cells by TMZ and TMZ@Astro‐exo‐FA. [file KJM2-40-435-s001.docx]

**Table S2** **Sensitivity of glioma and other non-glioma tumor cells by TMZ and TMZ@Astro-exo-FA**

| Cancer Type | Cell Line | TMZ IC_50_ | TMZ@Astro-exo-FA IC_50_ |
| --- | --- | --- | --- |
| Glioma | U251 | >50 μM | 14.3 μM |
| Glioma | U87 | >50 μM | 18.3 μM |
| Glioma (TMZ-R) | T98G | >1500 μM | 40.8 μM |
| Lung | A549 | >100 μM | >100 μM |
| Prostate | DU145 | >100 μM | >100 μM |
| Pancreas | PANC-1 | >100 μM | >100 μM |
| Breast | MDA-MB-231 | >100 μM | >100 μM |
| Melanoma | MDA-MB-435 | >100 μM | >100 μM |
| Colorectal | HT-29 | >100 μM | >100 μM |
