# Supplementary material for: Folic acid‐decorated astrocytes‐derived exosomes enhanced the effect of temozolomide against glioma
Source: Kaohsiung J Med Sci. 2024 Mar 14;40(5):435–44. doi: 10.1002/kjm2.12819 (PMC11895587; doi:10.1002/kjm2.12819)
Supplement: Supplementary file 3 — Table S3. The biochemical parameters in mice after different treatments values are mean ± SD for six mice in each group. ALP, alkaline phosphatase; ALT, alanine transaminase; AST, aspartate aminotransferase; BUN, blood urea nitrogen; Cre, creatinine. (L) represents a lower than 25% change from the untreated baseline, and (H) represents higher than 25% change from the untreated baseline. [file KJM2-40-435-s002.docx]

**Table S3** **The biochemical parameters in mice after different treatments**

|  | Control | TMZ | TMZ@Astro-exo | TMZ@Astro-exo-FA |
| --- | --- | --- | --- | --- |
| ALP (U/L) | 102.71±18.26 | 71.33±11.46 (L) | 82.19±3.87 | 90.89±12.98 |
| ALT (U/L) | 36.67±3.44 | 58.27±16.73 (H) | 31.06±3.31 | 36.74±1.99 |
| AST (U/L) | 198.35±47.77 | 331.59±84.18 (H) | 146.89±25.15 | 177.18±24.26 |
| BUN (mg/dL) | 16.78±1.86 | 20.74±1.24 (H) | 19.44±2.21 | 16.93±1.09 |
| Cre (mg/dL) | 0.15±0.08 | 0.15±0.05 | 0.16±0.05 | 0.13±0.02 |

Values are mean ± SD for six mice in each group. ALP: alkaline phosphatase, ALT: alanine transaminase, AST: aspartate aminotransferase, BUN: blood urea nitrogen, Cre: creatinine. (L) represents a lower than 25% change from the untreated baseline, and (H) represents higher than 25% change from the untreated baseline.
